# Neural Cell Chip Based Electrochemical Detection of Nanotoxicity

**DOI:** 10.3390/nano5031181

**Published:** 2015-07-02

**Authors:** Md. Abdul Kafi, Hyeon-Yeol Cho, Jeong Woo Choi

**Affiliations:** 1 Department of Microbiology and Hygiene, Bangladesh Agricultural University, Mymensigh-2202, Bangladesh; E-Mail: makafi2003@gmail.com; 2 Interdisciplinary Program of Integrated Biotechnology, Sogang University, Seoul 121-742, Korea; 3 Department of Chemical and Bimolecular Engineering, Sogang University, Seoul 121-742, Korea; E-Mail: yeol@sogang.ac.kr

**Keywords:** nanotoxicity, electrochemical method, RGD nanostructure, neural cell chip

## Abstract

Development of a rapid, sensitive and cost-effective method for toxicity assessment of commonly used nanoparticles is urgently needed for the sustainable development of nanotechnology. A neural cell with high sensitivity and conductivity has become a potential candidate for a cell chip to investigate toxicity of environmental influences. A neural cell immobilized on a conductive surface has become a potential tool for the assessment of nanotoxicity based on electrochemical methods. The effective electrochemical monitoring largely depends on the adequate attachment of a neural cell on the chip surfaces. Recently, establishment of integrin receptor specific ligand molecules arginine-glycine-aspartic acid (RGD) or its several modifications RGD-Multi Armed Peptide terminated with cysteine (RGD-MAP-C), C(RGD)_4_ ensure farm attachment of neural cell on the electrode surfaces either in their two dimensional (dot) or three dimensional (rod or pillar) like nano-scale arrangement. A three dimensional RGD modified electrode surface has been proven to be more suitable for cell adhesion, proliferation, differentiation as well as electrochemical measurement. This review discusses fabrication as well as electrochemical measurements of neural cell chip with particular emphasis on their use for nanotoxicity assessments sequentially since inception to date. Successful monitoring of quantum dot (QD), graphene oxide (GO) and cosmetic compound toxicity using the newly developed neural cell chip were discussed here as a case study. This review recommended that a neural cell chip established on a nanostructured ligand modified conductive surface can be a potential tool for the toxicity assessments of newly developed nanomaterials prior to their use on biology or biomedical technologies.

## 1. Introduction

Nanoparticles are widely used in many materials, consumables and biomedical technologies. However, their potential risk for animal, human and environments is still to be identified [1]. In recent decades, nano-structured materials or biomaterials or nano-biomaterials have shown great promise for advancing the field of biology and medicine [2,3,4]. Like nano organisms (viruses), the precise structure and size of the nanomaterials allow their readily exposure to sub cellular structures including genomic systems, and even to the central nervous system of living beings, which ultimately result in significant health hazards [5,6,7,8]. Hence, the assessment of nanoparticle’s toxicity prior to use in the daily consumable products or nano-biotechnologies is of great scientific and public health interest. Several spectrophotometric *in vitro* cytotoxicity assays using biochemical methods such as 3-(4,5-dimethylthiazol-2-yl)-2,5-diphenyltetrazolium bromide test (MTT), neutral red uptake (NRU), Adenosine triphosphate (ATP) and lactate dehydrogenase (LDH) measurement, sulforhodamine B (SRB) assay, water-soluble tetrazolium salt-1 (WST-1) assay used for nanotoxicity assay [9,10,11]. The conventional spectrophotometric assay-based analytical methods of toxicity assessment are usually time-consuming process and often lack sensitivity resulting in inaccurate data due to photo bleaching [12,13,14]. Therefore, researches have focused on a label free *in situ* detection of cellular physiologic response to any toxicant or physical or chemical stimulus, which has attracted considerable attention of bioengineers [12,13,14,15,16,17]. In the recent past, acetylcholinesterase (AChE)-inspired biomimetic sensor was employed to accurately predict toxicity of AChE inhibitors and overcomes the short shelf-life and portability issues of current biological assays [18]. Recently, an electrochemical monitoring of cellular behavior has proven to be a fast, accurate and sensitive tool [15,16,17].

The living whole cell-based chip is considered to be potential tool for the precise, accurate, sensitive measurements of toxicity [19,20]. In the cell based assay, the living cell is employed as a detector of analytes from environment, food, chemical, or other sources [12,13,21,22,23]. Therefore, cell chips are the emerging alternative to other analytical methods for nanoparticle’s toxicity detection. The main advantage of using tissue specific cell chips for probing toxicity of various nanomaterials is that cell chips respond to the material exposure in the manner related to the actual physiologic responses of the vulnerable subjects [15,16,17,24,25]. The results obtained from cell chip are based on the nanoparticle-cell interactions, and therefore, reveal functional information about the particle [15,17]. Considering the sensitivity of neural cell to the external influences like environmental toxicant, anticancer agent, cytotoxic chemicals, neural cell based chip can be the most suitable for the nanoparticle’s toxicity determination [12,13,26,27]. Neural cell chip encompasses immobilization of a neuron on a conductive surface and an electrical transducer in which cellular responses to the external stimuli of toxicant accumulated from the cell electrode interfaces [12,15,17,28]. Hence, the toxic response of a nanoparticle has been recorded accurately and sensitively from a neural cell based chip [29,30,31]. However, a detail discussion on the fabrication strategies and use of neural cell based chips with particular emphasis on their limitation and opportunity is needed to improve this emerging tool for accurate and effective nanotoxicity assessment.

Therefore, this review discusses several neural cell chip based researches sequentially to overcome the limitations that arise during their designing, fabrication and electrochemical measurements. In addition, as a case study, the effective monitoring of nanotoxicity of quantum dot (QD), graphene oxide (GO) and cosmetic compounds have been discussed here to prove the potentiality of the neural cell chip. This article recommends that a neural cell immobilized on arginine-glycine-aspartic acid (RGD) nanostructures modified conductive materials surface is a very effective tool for the characterization and/or absolute quantification of engineered nanomaterial and nanotoxicity.

## 2. Nanotoxicity Assessment Based on Electrochemical Methods

**Figure 1:**
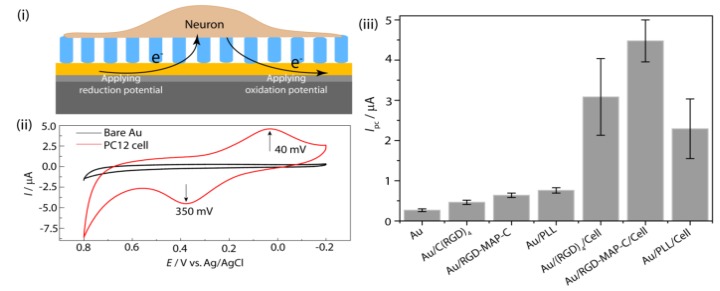
Schematic representation of redox phenomena at cell-substrate interface (**i**) and the redox peaks obtained from PC12 cell immobilized on peptide fabricated Au electrode (**ii**); (**iii**) cyclic voltammetry (CV) for comparing the electrochemical signal using C(RGD)_4_, Arginine-Glycine-Aspartic acid-Multi Armed Peptide terminated with Cysteine (RGD-MAP-C) and poly-l-lysine (PLL) fabricated Au electrode without PC12 cell. Reproduced with permission from [28], Copyright 2010, Elsevier.

A living cell immobilized on a metal electrode surface exhibits a distinct redox activity by exchanging electron between the cell-electrode interfaces [25,28]. This electron exchange phenomena is detected by electrochemical apparatus, represents cellular electrophysiological state [15,16,17]. Figure 1 shows the redox behavior of neural cell immobilized on peptide modified Au surface. This electrophysiological state is strictly influenced by any electrical, chemical or toxic stimulus [12,13,14,32]. Based on this phenomenon, redox signals obtained from various toxins exposed cells can be translated in to the cell viability which are excellent indicators for assessing toxicity of a material [13]. Almost all materials including drugs, chemicals, biological materials and even nanoparticles are the candidates whose toxicity can be assessed by cell chip based on electrochemical tools [17,25,33,34]. However, this emerging tool possesses some technical challenges, particularly proper adhesion of cell on electrode surface, appropriate design of chip chamber and perfect electrochemical recording using cell line specific redox windows [15,16,28]. A few recent studies have shown how to overcome these limitations and use this tool for accurate and sensitive monitoring nanotoxicity of emerging nanomaterials of interest [15,16,17,28,35].

*Effective immobilization of neuron on electrode surfaces:* Establishment of neuron cell on a metal electrode surface is difficult due to their weak or loose attachment tendency [36,37]. Several studies reported that incorporation of artificial ligand on the chip surface can overcome this shortcoming [17,28,38]. Several biopolymers like collagen or its components like RGD and poly-l-lysine were successfully incorporated with the chip surface for enhancing cell adhesion ability [17,28]. Among the extracellular matrix components, collagen was proven to be the most suitable for attachment on the chip surface and confer strongest cell adhesion [38]. However, the collagen modified chip is still facing difficulties during the electrochemical measurement [39]. Collagen forms a thick layer on the chip surface during the fabrication process that impaired the electron exchange phenomenon at the cell-electrode interface [40]. Later, it was well understood that only RGD enriched portion of collagen molecule is involved in the cell attachment process through RGD-integrin coupling method [41]. Therefore, our group focused on the use of RGD tripeptide sequence on the cell chip for enhancement of cell attachment [15,28,35].

**Figure 2:**
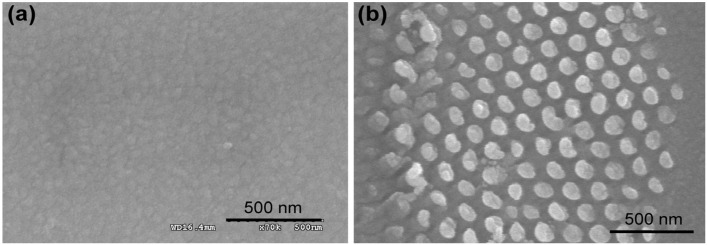

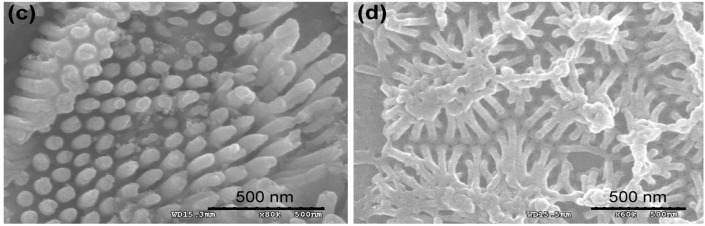
Representative scanning electron microscopy (SEM) images of the Au surfaces employed in the cell experiments. The images obtained at 20 Hz from the sample pre-coated with Pt-Pd alloy. (**a**) Freshly cleaned bare Au surface prior to peptide immobilization; (**b**) 2D-RGD nanodots; (**c**) 3D-RGD nanorods; and (**d**) 3D-RGD nanopillars array. Scale bar 500 nm. Reproduced with permission from [35], Copyright 2012, Elsevier.

**Figure 3:**
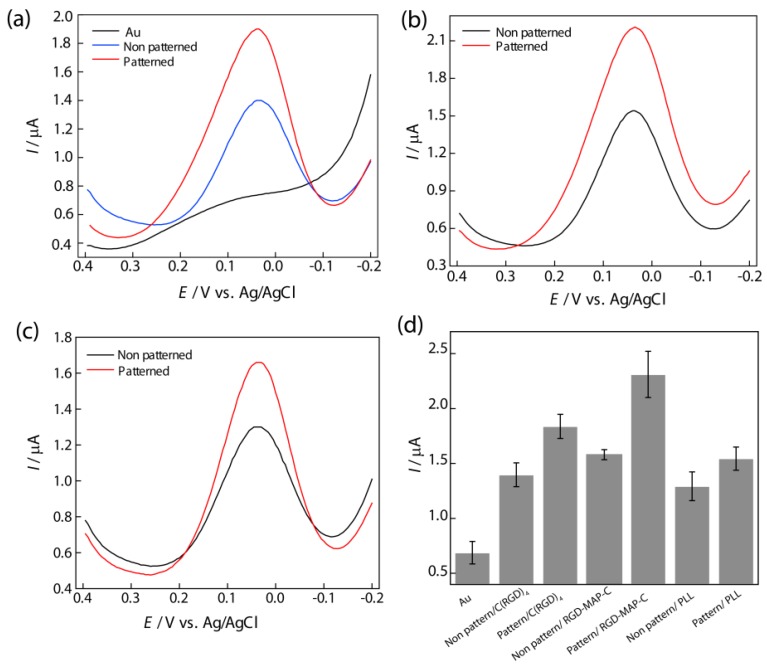
Differential pulse voltammogram of PC12 cell to compare the effects of patterned and non-patterned peptides on Au electrode, (**a**) C(RGD)_4_; (**b**) RGD-MAP-C; (**c**) PLL using phosphate buffer saline (PBS, 0.01 M pH 7.4) as an electrolyte at a scan rate of 100 mVs^−1^. Pulse amplitude and pulse width adopted were 50 mV and 50 ms, respectively; (**d**) Comparison between DPV peak current from PC12 cells on the different peptide fabricated Au surface. Data are the mean ± standard deviation of three different experiments. Reproduced with permission from [28], Copyright 2010, Elsevier.

A nanoscale thin film of RGD tripeptide sequences was found to be suitable for neural cell immobilization as well as for electrochemical measurement [12,42,43]. The cysteine terminated RGD tripeptide sequences coupled with integrin receptor of cell membrane confers farm attachment between cell and electrode [28]. The nano-scale thin-film of RGD peptide was achieved by the self-assembling of cysteine terminated peptide residue, where cysteine terminated end strongly attached with Au surface through thiol-gold coupling method [15,33]. Later, RGD peptide was nano-patterned on the Au surface at a defined nanoscale alignment using the mask guided self-assembly method [35]. Several nano-archistructural arrangements of RGD peptide were also achieved using spatially size controlled porous mask at defined concentration and dose (Figure 2). Practically, the nano-patterned RGD modified surface showed better cell adhesion and proliferation as well as electrochemical measurements (differential pulse voltammetry, DPV) compared to their homogenous thin-film like arrangement (Figure 3). In addition, among the nanostructured RGD, the three dimensional arrangement was found to be most suitable for cell functions compared to their two dimensional or thin biofilm like arrangement [25].

**Figure 4:**
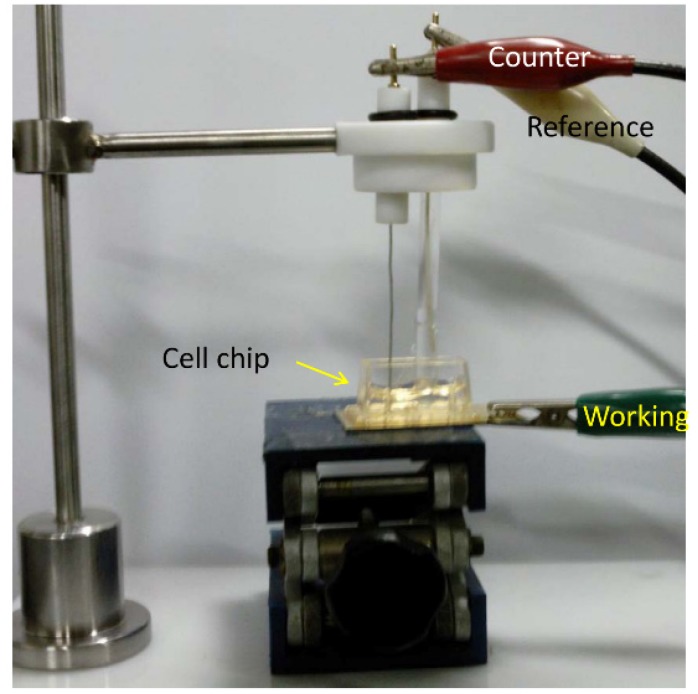
The fabricated chip with thee electrode setup for electrochemical measurement of cell cycle progression. Adapted with permission from [15], Copyright 2011, American Chemical Society.

*Cell chip chamber design and electrochemical setup:* Silicon based Au electrode is commonly using in the cell chip fabrication, where a 50 nm thick titanium (Ti) layer is established on the silicon substrate and then a 150 nm thick gold (Au) layer is patterned by DC magnetron sputtering [44]. The Au surface was cleaned with piranha solution as previously described elsewhere [12,15,17,28]. Then it was polished carefully by sonication in absolute alcohol and double-distilled water for 5 min, respectively. Finally, the electrode was electrochemically cleaned in 0.5 M H_2_SO_4_ until a stable cyclic voltammogram was obtained and dried with purified nitrogen [15]. The substrate was washed with deionized distilled water and dried under N_2_ gas. Then, RGD tripeptide sequences were established on the Au surface as discussed before (Effective immobilization of neuron on electrode surfaces chapter) [35]. Finally, a chip chamber was achieved by affixing a plastic chamber of a definite dimension with polydimethylesyloxane (PDMS) on substrate (Figure 4). We observed that chip chamber (Lab-Tek^®^, Thermo Fisher Scientific, Waltham, MA, USA) of 2 cm × 1 cm × 0.5 cm (width × length × height) dimensions established on a freshly prepared Au working electrodes with an area of 3 cm^2^ is suitable enough for electrode placement as well as for electrochemical measurement [28].

The electrochemical measurement was impaired by the exposure of environmental oxygen, which was usually prevented by the continuous flow of N_2_ gas during the measurement. Therefore, the proposed designed chip with 2.6 mm^2^ exposure area for cell attachment overcomes environmental oxygen exposure by the constant flow of N_2_ gas [15]. Electrochemical measurements were performed with a three-electrode setup, where a freshly prepared chip with cell was used as a working electrode and platinum (Pt) and silver (Ag/AgCl) as counter and reference electrode, respectively (Figure 4).

**Figure 5:**
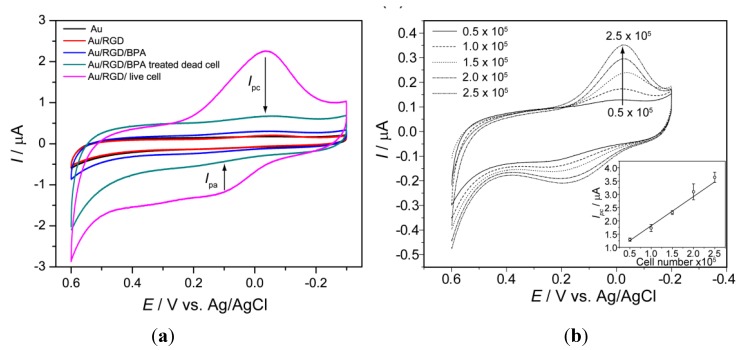
Characterization of chip: (**a**) Cyclic voltammograms from bare Au, Au/RGD, Au/RGD/BPA, Au/RGD/SH-SY5Y(with BPA) and Au/RGD/SH-SY5Y(without BPA) cells immobilized on an Au chip, (↓) indicates corresponding decreased peak due to 2 µM BPA treatment; (**b**) Cyclic voltammogram for SH-SY5Y cells with different numbers, (↑) arrow indicate current increased with cells number and inset shows linear plot of cathodic peak current (*I*_pc_) as a function of cell number, *R*^2^ = 0.99. The voltametric measurements were performed using PBS (0.01 M pH 7.4) as an electrolyte at a scan rate of 100 mVs^−1^. Reproduced with permission from [12], Copyright 2011, Elsevier.

*Recording of cell line specific peak potential:* Electrochemical analysis of a cell immobilized on RGD modified [35] chip reveals potential of anodic (*I*_pa_) and cathodic peak current (*I*_pc_), which is used as a parameter for quantifying cell numbers as well as viability of the cells against any potential environmental toxicant [13]. These potential peaks showed cell line specificity [16]; therefore, the cell to be used for the toxicity determination should be pre-checked its potential windows which will be applied during electrochemical measurements. PC12 cell (a neuro progenitor cell line) gives cathodic peak at 0.40 V and anodic peak at 0.390 V (Figure 1ii) during CV measurement while being investigated using three-electrode configuration with standard silver (Ag/AgCl) as the reference and a platinum wire as the counter electrode. In contrast, a well-defined redox peak was recorded when cyclic voltammetry (CV) was performed to analyze the electrochemical behavior of SH-SY5Y (neuroblastoma) cells on the home-made Au chip in the potential range of −0.3 V to 0.6 V (*versus* Ag/AgCl) at a scan rate 100 mVs^−1^. A quasi-reversible process with a *I*_pc_ at −0.056 V Ag/AgCl and an *I*_pa_ at 0.180 V Ag/AgCl, was clearly observed in cyclic voltammogram which was not detected form BPA treated dead cells or bare electrode without cell (Figure 5).

**Figure 6:**
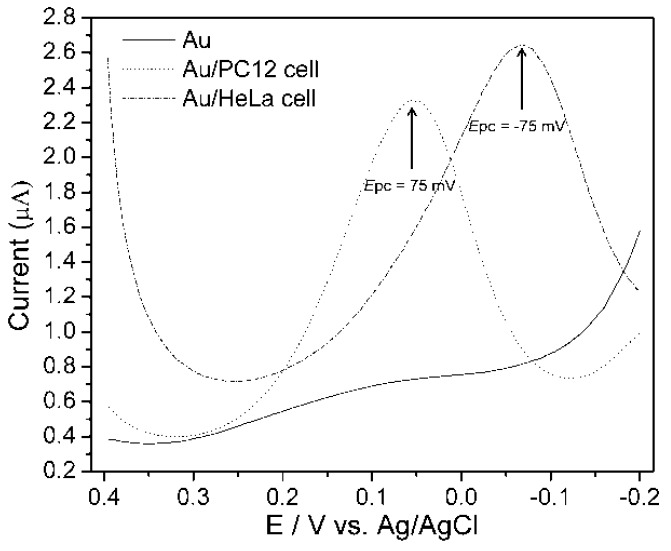
Differential pulse voltammogram (DPV) of PC12 and HeLa cell on collagen modified Au surface. DPV was measured using PBS (0.01 M, pH 7.4) as an electrolyte at a scan rate of 100 mVs^−1^. Pulse amplitude and pulse width were 50 mV and 50 ms, respectively. Reproduced with permission from [16], Copyright 2011, IARIA.

An experiment considered the anodic peak potential that was obtained from CV technique at a potential window of −0.2 V to 0.4 V that was applied to measure DPV from PC12 and HeLa cell lines at a scan rate of 100 mVs^−1^, with 50 mV pulse amplitude and 50 ms pulse width. Well distinguished DPV signals were measured from PC12 and HeLa cell [16]. Figure 6 shows that a PC12 cell gives the peak at 75 mV and HeLa cell at −75 mV, whereas no peaks were observed from bare Au surface, indicating that peaks certainly appear from the cells when they were immobilized on the Au electrode surfaces. Therefore, the cell line specific electrochemical signals were proven by both (CV and DPV) of the amperometric methods.

## 3. Electrochemical Toxicity Assessment of Quantum Dot (QD)

**Figure 7:**
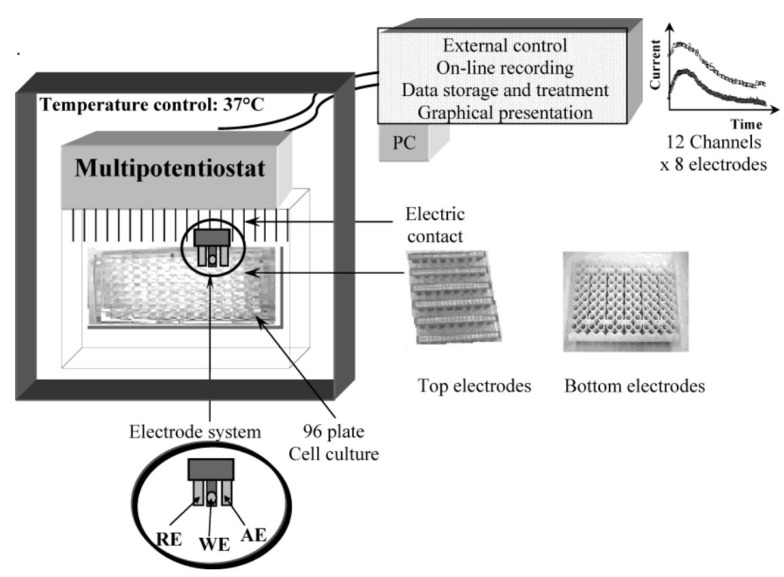
Schematic diagram of the multi-channel dissolve oxygen (DO_*x*_) sensor system used for measuring cytotoxicity. Two configurations of the 96 electrodes are available with the electrodes at the top or bottom of the wells. Each sensor consists of three electrodes: reference (RE), auxiliary (CE), and working (WE) electrode. Reproduced with permission from [50], Copyright 2004, American Chemical Society.

Despite the numerous beneficial applications, the potential health effect of QD cannot be ignored. The smaller particle size of QD allows its ready exposure throughout the body, including the central nervous system [45,46,47]. Therefore, health hazard analysis is urgently needed to assess their possible toxicity prior to its application in biomedical technologies or consumables. Traditionally, toxicity of a particle is usually assessed by determining cytotoxicity based on MTT, XTT, fluorescence and trypan blue techniques [9,10,11]. In the recent past, nanotoxicity was assessed by dissolve oxygen sensor (DO_*x*_ sensor), which is a prototype multichannel system capable of simultaneous, quantitative and continuous measurement of dissolve oxygen [48]. The DO_*x*_ sensor is a fully automated, porTable 96-well electrode biosensor equipped with a multi-potentiostat that employs a 96-well electrode biosensor known as the DO_*x*_-96 device (Figure 7). Its experimental setup involves use of 96 disposable electrodes in a standard three-electrode system (reference, working and counter) where reduction in the dissolved oxygen due to cellular respiration is determined electrochemically as amperometric current [49]. The rates of oxygen consumption by cell immobilized electrodes indicate their viability rates [50,51].

**Figure 8:**
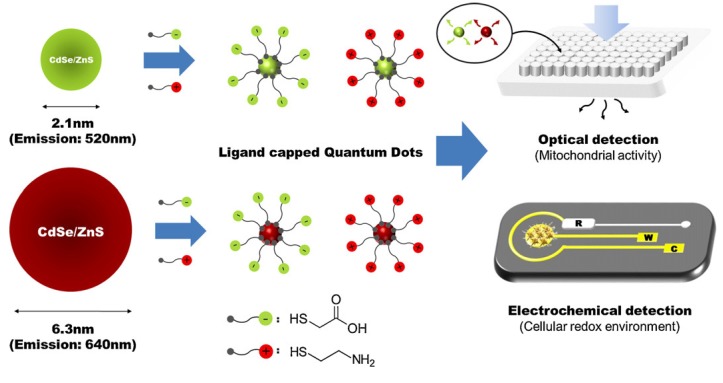
Schematics for the detection of cytotoxicity of thioglycolic acid (TA) or cysteamine (CA)-capped green and red quantum dots (QDs) based on cell chip and conventional MTT assay. “R”, “W” and “C” mean the reference, working, and counter electrodes, respectively. Reproduced with permission from [29], Copyright 2012, Elsevier.

The potential DO_*x*_ based cell viability assay was further employed for the detection of cytotoxicity of Zn/Te quantum dot [50]. For this, cell culture plates were maintained in standard cell culture incubator at 37 °C under 5% CO_2_ and humidified chamber, and reduction current was measured for 30 min under a constantly applied potential (400 mV). The current peaks obtained from quantum dot treated cell culture plate were compared with current peaks obtained from control culture plate (without QD treated). The QD treated cells showed dose dependent increase in current inequities indicating that Zn/Te QD causes cytotoxicity resulting reduced oxygen consumption. However, this method requires a smooth optimization protocol which will insure a sterility of system with desired CO_2_ and temperature level, electrode repitability and suitable application potential. Very recently, our group introduced a simple, highly sensitive cell-chip based electrochemical tool for the toxicity assessment of various chemicals, environmental toxicants or nanomaterials [12,13,26,27]. A neural cell-chip was fabricated on RGD-MAP-C modified Au surface to assess toxicity of CdSe/ZnS on intracellular redox environment SH-SY5Y cell [29]. The SH-SY5Y cell immobilized working electrode were established on a silicon surface by DC magnetron sputtering and a platinum and standard silver electrode were also place as counter and reference, respectively (Figure 8). The electrochemical measurements were performed using CHI potentiostat instrument; the *I*_pc_ and *I*_pa_ from SH-SY5Y cell were measured at 250 mV and 365 mV, respectively. Variations of current peak intensities from CdSe/ZnS treated and non-treated SH-SY5Y cell were considered as a parameter for the cytotoxicity assessment.

**Figure 9:**
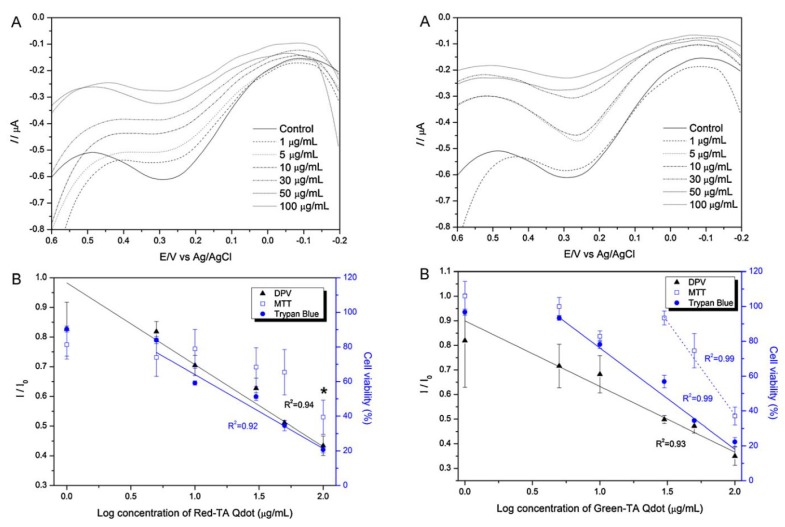
Concentration-dependent effects of Red-TA QDs on SH-SY5Y cells: (**A**) Differential pulse voltammogram of SH-SY5Y cells treated with different concentrations of Red-TA QDs (1, 5, 10, 30, 50, and 100 g/mL); (**B**) Correlations between cell viability and DPV, MTT, and trypan blue assay results corresponding to different concentrations of Red-TA QDs. *I*_0_ means the peak current obtained from cells not treated with Red-TA QDs. Huge decrease of cell viability are marked with an asterisk (*). Error bars are the mean ± standard deviation of three different experiments. Reproduced with permission from [29], Copyright 2012, Elsevier.

Using the neural cell based chip nanotoxicity of 100 nm-sized silica nanoparticles (SNP) were evaluated on SH-SY5Y cells using CV method. The current peaks obtained from SNP treated cell were found to exhibit reverse relation with doses of nanoparticles. SNP was found to show mild toxicity at dose of 50 μg/mL and acute toxicity at 200 μg/mL that decreased the current intensity as almost 70% *versus* control group [52]. Toxicity of Green and red-emitting CdSe/ZnS QDs were also evaluated using the same electrochemical setup (Figure 9). Where, *I*_pc_ values were measured from the SH-SY5Y cell at a potential of 210 mV and used as an indicator for determining nanotoxicity. SH-SY5Y cell was found to exhibit various degree of toxicity when exposed to the Green- and Red emitting (6.3 nm in diameter) QDs separately. The Red emitting QD showed a toxicity five times more that green emitting (2.1 nm in diameter) QD.

The cytotoxicity of ligand-capped CdSe/ZnS QDs was also confirmed by another ampere-metric method, DPV. In this case, a concentration dependent negative linearity in peak current was achieved as illustrated in Figure 9B. The dose dependent potential toxicity were further assessed by the traditional trypan blue exclusion assay as well as MTT viability assays to compare the potentiality of neural cell chip based toxicity analysis. MTT assay reveals the potential toxicity of Red-TA QDs and Green-TA QDs at dose of 100 g/mL and 50 g/mL, respectively, while electrochemical measurements reveals that DPV signals start to decrease at a dose of 10 mg/mL and 5 mg/mL, respectively. So, electrochemical measurements reveal a similar trend of QDs toxicity but more sensitively compared to traditional MTT bases toxicity assay. The electrochemical measurement of QDs toxicity was further verified with the trypan blue based microscopic exclusion method. The negative linearity of cell numbers obtained from various concentrations of Red-TA and Green-TA QDs is consistent with the linearity of current peaks obtained from DPV measurements. The decreased cell viability recorded from various doses of Red-TA and Green-TA QDs treatments were more accurate by the electrochemical method compared to traditional trypan blue based exclusion method or MTT based mitochondrial reductase activity assay. This indicated that intracellular redox environment is more sensitive than other components (mitochondria, cell membrane) and can be a useful indicator for the assessment of cytotoxicity of nanoparticles which have potential toxicity even at very low concentrations. Hence, the recently developed neural cell chip can be applied to assess nanotoxicity of various engineered nanomaterials with high accuracy, reliability, and reproducibility.

## 4. Electrochemical Toxicity Assessment of Graphene Oxide (GO)

**Figure 10:**
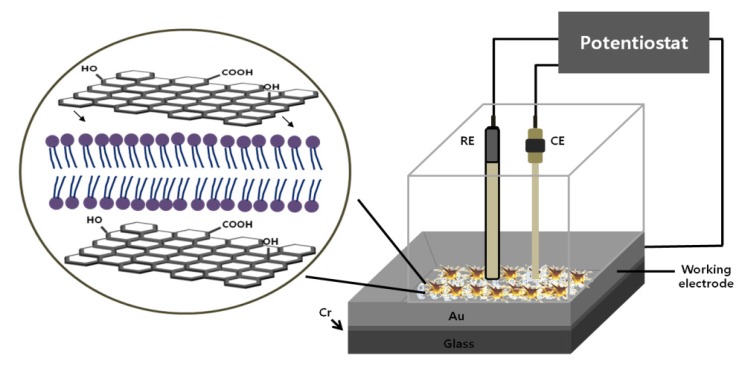
Schematics for the electrochemical detection of the toxic effect of Graphene oxide (GO) on human neural stem cell (HB1.F3). Reproduced with permission from [30], Copyright 2012, American Scientific Publishers.

Graphene and its derivatives become promising candidates for numerous important biomedical applications because of their excellent conductivity, high optical transparency and considerable rigidity [53,54,55]. GO is an insulating material that contains many hydroxyl groups on its surface suitable for drug carriers and/or photo thermal therapeutic agents [56,57]. However, their practical applications in biology or biomedical technologies require detailed comprehension of toxicity. The possibility of GO exposure to human and animal increases due to rapid expansion of nanotechnologies which is likely to be several-fold in the future. Several studies have been undertaken to assess the effect of biological interactions of GO on different organizational levels of living system, from biomolecules to animals [29]. This review focuses on electrochemical assessment of *in vitro* nanotoxicity of GO based on neural cell chip. A neural cell chip fabricated on Au surface functionalized with RGD peptide using HB1.F3 cell as a potential neural candidate was the first approach to determine GO toxicity assay. A freshly prepared HB1.F3 cell immobilized chip was exposed to GO nano-pellet for 12 h prior to electrochemical measurements (Figure 10). Three-electrode system CV and DPV were performed using a potentiostat (CHI-669, CHI, Austin, TX, USA) controlled by general purpose system software. Where, HB1.F3 cell immobilized Au electrode served as working and platinum and Ag/AgCl serves as counter and reference electrode respectively.

**Figure 11:**
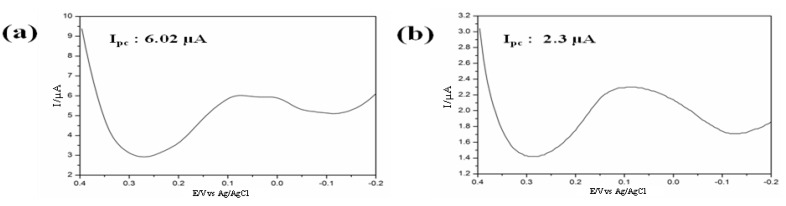

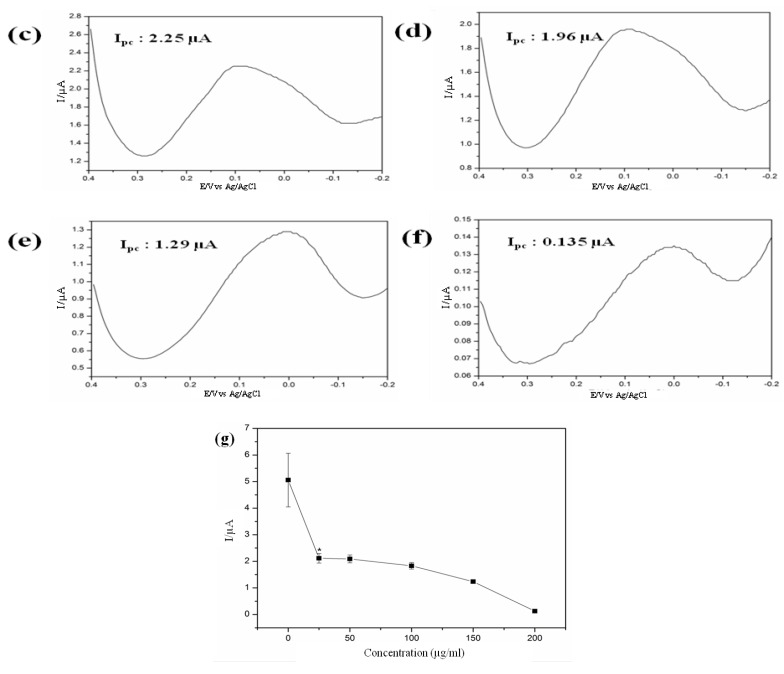
Electrochemical responses of HB1.F3 cell treated with (**a**) 0 μg/mL; (**b**) 25 μg/mL; (**c**) 50 μg/mL; (**d**) 100 μg/mL; (**e**) 150 μg/mL and (**f**) 200 μg/mL of GO nano-pellets obtained via DPV; (**g**) Decreases in the peak current of the DPV signal that corresponded to different concentrations of GO nano-pellets with which the HB1.F3 cells were treated (* *p* < 0.05 *vs*. the control; the error bars stand for the standard deviation of three different experiments. Reproduced with permission from [30], Copyright 2012, American Scientific Publishers.

The electrochemical measurement reveals that HB1.F3 cells gave oxidation (*E*_pa_) and reduction (*E*_pc_) peak at 0.00 V and 0.35 V respectively [30]. Later, the reduction peak current (*I*_pc_) were determined by DPV using a potential window of −0.2 V to 0.4 V with a 0.05 V amplitude and a 0.05 s pulse width. The intensities in current peaks obtained from various doses of GO nano-pellets treated electrodes were analyzed to determine the cytotoxicity of GO. Figure 11 reveals that current peaks decreased significantly when 25 μg/mL GO nano-pellets were applied and followed by continued decreasing tendency with the increased doses. This features of current peaks completely coincided with the cell viability data based on MTT assay indicating the validity of neural cell chip based electrochemical monitoring of GO nanotoxicity assay. The acute toxicity of GO even at low concentration (25 μg/mL) to human neural cell is confirmed by both the recently developed electrochemical as well as traditional MTT based method. Therefore, considering the accuracy and sensitivity, a neural cell based chip can be employed for assessing the toxicity of newly developed nanomaterials prior to their field applications.

## 5. Electrochemical Detection of Nanotoxicity of Cosmetic Compound

A wide variety of nano-structured materials are now used in daily commodities, pharmaceutics, cosmetics, biomedical products, and industries [58,59,60,61]. Most of the famous cosmetic companies have been registered numbers of patents of nanoparticle based products since the last decade [61]. However, these nanoparticles based cosmetic products can be potential risks for dermal nanotoxicity through direct exposure. Macro, micro, or submicroscopic abrasion by any means represents an excellent port of entry for nanoparticles [1,45]. In addition, their subsequent translocation through the lymphatic channel followed by blood circulation allows their exposure throughout the body, which might result in generalized toxicity [1,45]. Therefore, nanoparticle’s toxicity assessment has enormous significance prior to their incorporation in the cosmetic products. For the first time, our group has introduced dermal fibroblast cell based chip for electrochemical determination of nanotoxicity of cosmetic compounds [31].

**Figure 12:**
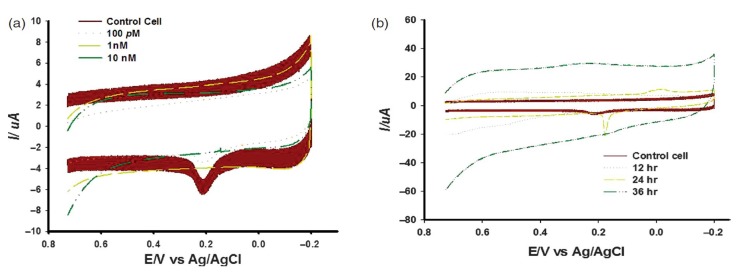
Effect of imidazolidinyl urea on skin fibroblast cells, (**a**) Cyclic voltammogram for skin fibroblast cells treated with different doses of imidazolidinyl urea; (**b**) Cyclic voltammogram for skin fibroblast cells detected at different times after imidazolidinyl urea treatments. Reproduced with permission from [31], Copyright 2012, American Scientific Publishers.

In the first approach, we have investigated imidazolidinyl urea induced cytotoxicity on human fibroblast cells (HFF cells) based on electrochemical method. For this, a Gold nanostructured surface was fabricated by Mask Guided Assembly (MGA) method using a nanoporous alumina mask synthesized by two steps annodization protocol. For the effective immobilization of cell, the Au nanostructured surface was further functionalized with RGD peptide sequences as mentioned in previous chapter. Then, HFF cells were allowed to attach to RGD functionalized nano-patterned Au surface, and three-electrode system electrochemical tool was applied to the chip surface for monitoring cellular influences to the imidazolidinyl urea. It is well known that HFF cells produce an inflammatory response to allergens like- imidazolidinyl urea. Therefore, the HFF cell treated with different concentrations of imidazolidinyl urea for 24 h should obviously be induced various degrees of inflammatory responses. These dose dependent inflammatory responses were successfully monitored using voltammetric measurement of peak current (Figure 12a). The electrochemical data demonstrated that imidazolidinyl urea significantly reduces current peaks in a time and dose-dependent manner (Figure 12b). This showed that the current peak was reduced in accordance with the increases in imidazolidinyl urea induced inflammation. Therefore, this study suggested that an electrochemical-based chip provides crucial information for improvements to a cell chip system for nanotoxicity screening. Hence, the proposed HFF cell based chip can be a suitable tool for testing of nanoparticle based cosmetic compounds before their practical use.

## 6. Conclusions

Owing to the potential tunable properties, nanomaterials application is increasing in the daily commodities including biology, biomedical technologies and even in several blooming businesses like cosmetic industries. Assessment of potential health effects of engineered nanomaterial has become important step towards creating safety guidelines for their handling and disposal. In the present review, we discussed the nanotoxicity assessment based on newly developed neural cell chip technologies with an emphasis on their fabrications and *in situ* measurement techniques. The detailed discussion suggested that this emerging tool possesses some technical challenges, particularly, the proper adhesion of cell on an electrode surface, appropriate design of chip chamber and efficient electrochemical recording using cell line specific redox window. A series of recent studies have recommended some measures to overcome these limitations and to improve this tool for accurately and sensitively monitoring nanotoxicity of emerging nanomaterials. According to this review, a neuron cell should sufficiently be attached on a conductive material surface which is achieved by RGD functionalization. A plastic chamber of 2 cm length × 1 cm height × 1 cm width should be affixed on the RGD functionalized surface which is sufficient enough for electrode placement and effective electrochemical measurements by preventing atmospheric O_2_ exposures using controlled N_2_ flow. Cell line specific potential windows should be predetermined prior to their use as a cell chip for nanotoxicity determination because cellular redox potential varies from cell line to cell line. Finally, successful monitoring of QD, GO and cosmetic compound toxicity using this newly introduced neural cell chip were discussed here as case study. The RGD nanostructures modified neural cell chips were found to be most suitable method for the accurate, sensitive, *in situ* monitoring of engineered nanoparticles toxicity. However, electrochemical measurement of the current design chip in open air are still facing challenges in accurate and sensitive monitoring of the cellular responses. Proper design and fabrication of the chip chamber like a small scale automated bioreactor can overcome this limitation and certainly influence effective monitoring of nanotoxicity of an engineered material. However, considering the sensitivity and conductivity of a neural cell, the proposed chip can be a potential tool for the assessment of health risk of nanomaterials to suggest a safety guideline for their handling and disposal.
